# Curative care expenditure of outpatient anxiety disorder in Liaoning Province, 2015-2020-based on “System of Health Accounts 2011”

**DOI:** 10.3389/fpubh.2024.1329596

**Published:** 2024-07-03

**Authors:** Xiaoxia Shi, Yue Zhao, Quan Wan, Peipei Chai, Yuedan Ma

**Affiliations:** ^1^Department of Traditional Chinese Medicine, School of Graduate Students, Liaoning University of Traditional Chinese Medicine, Shenyang, China; ^2^China National Health Development Research Center, Beijing, China; ^3^Department of Public Management, School of Economics and Management, Liaoning University of Traditional Chinese Medicine, Shenyang, China

**Keywords:** anxiety disorder, outpatient expenditure, CCE, SHA2011, burden

## Abstract

**Introduction:**

Anxiety disorders are the most common mental disorder, experienced by more than a quarter of the population. This study examines total outpatient curative care expenditures (CCE) for anxiety disorders and changes in their composition based on the System of Health Accounts 2011 (SHA 2011).

**Methods:**

This study used multi-stage stratified random from a total of 9,318,513 outpatient sample data by 920 healthcare organizations, a total of 109,703 cases of anxiety disorders from 53 sample organizations (5.76%) from 2015 to 2020. Univariate analysis, multifactor analysis and structural equation modeling (SEM) were used to explore the influential factors affecting outpatient CCE for anxiety disorders.

**Results:**

Anxiety disorder outpatient CCE from 2015 to 2020 continued to increase from CNY 99.39million in 2015 to CNY 233.84 million in 2020, mainly concentrated in western medicine costs, 15–64 years, general hospital, generalized anxiety disorder and public financing. The results of univariate analysis showed statistically significant differences in all subgroups, and the results of multivariate analysis and SEM showed that the choice to purchase western drugs, purchase prepared Chinese drugs, choice to have a checkup, urban employees’ basic medical insurance, and 0–14 years old were associated with high anxiety disorder outpatient CCE.

**Conclusion:**

Initiatives to improve the essential drug system, reduce the out-of-pocket (OOP) ratio, and strengthen primary health care to effectively reduce the medical burden on patients.

## Introduction

Anxiety disorders are among the most common mental disorders worldwide (1), are a leading cause of disability and premature death (2), and are ranked by the World Health Organization as the sixth contributor to disability [3.4% of disability-adjusted life-years (DALYs) in 2015] (3). More than a quarter of the total population experiences anxiety disorders at some point in their lives (4), and the duration of the illness averages 23.4 months (5), the lifetime prevalence of anxiety disorders is 34.0%, with a lifetime prevalence of 13.0% for social anxiety disorder, 6.2% for generalized anxiety disorder, 5.2% for panic disorder, and 2.6% for agoraphobia (6). Due to their intense, persistent and recurrent fear, anxiety, and restlessness, often accompanied by a range of physical symptoms such as palpitations, chest tightness, dizziness, vertigo, and syncope (1), and may precipitate or exacerbate cardiovascular disease, gastrointestinal disorders, lung diseases, cancer, chronic pain, and migraine (7). Both the mental torment caused by anxiety disorders themselves and the utilization of outpatient and specialized health care services (1, 8), as well as their leading to reduced educational programs, impaired interpersonal relationships (9), unemployment, impaired personal functioning, and even suicide in patients, place a heavy burden on individuals, families, and society. Information on the direct medical financial burden of the disease is essential for healthcare policy makers attempting to reduce the financial burden of anxiety disorders, so it is important to estimate the direct financial burden of anxiety disorders (10).

Countries around the world have a large number of people with anxiety disorders. As of 2019, there were 301.39 million people with anxiety disorders worldwide, accounting for 31.07% of people with mental illnesses, with a prevalence of 4.05, 1.13% of DALYs for all disorders, and 3.34% of years lived with disability (YLDs) for all disorders (11). Anxiety disorders impose a significant economic burden on all countries. The direct cost of anxiety disorders is 2.08% of health care costs (12) and 0.25–0.78% of GDP (2). All other costs are projected to reach £14.2 billion by 2026, with healthcare costs reaching £2 billion (13).

Previous studies in China have focused on epidemiology (14, 15), examining the prevalence of anxiety disorders in different dimensions and in different provinces, and have not addressed anxiety disorder cost accounting and measured province-specific costs (16–23). Others focused on cost analyses of specific treatments [including cognitive-behavioral therapy (24–26), digital intervention therapies (27), and reception and commitment therapy (28)], randomized controlled trials (29), specific types of anxiety disorders [generalized anxiety disorder (30, 31)], treatment with medications (32, 33), and co-morbidities (2, 34, 35) were analyzed for costs. Although these studies help to promote the development of anxiety disorder cost analysis, there is currently no larger system representative sample or systematic accounting framework to account for anxiety disorder costs in a region, and having a systematic accounting framework to account for anxiety disorder costs can improve the accuracy of measurement and comparability between different countries.

For “Whether to purchase western drugs,” the grouping method was that the answer to the question of “Whether to purchase western drugs” was “Yes” for those who had western drug costs in the cost breakdown of outpatient visits for anxiety patients, and the answer to the question of “Whether to purchase western drugs” was “NO” for those who did not have western drug costs in the cost breakdown of outpatient visits for anxiety patients. The answer to the question “Whether to purchase western drugs” is “Yes” for those who do not have western drugs in the cost breakdown of anxiety disorder outpatient visits, and the answer to the question “No” for those who do not have western drugs in the cost breakdown of anxiety disorder outpatient visits. For Whether to purchase prepared Chinese drugs, the grouping method was that the answer to Whether to purchase prepared Chinese drugs was “Yes” for prepared prescription (Chinese medicine) and herbal medicines that existed in the cost breakdown of the outpatient clinics for anxiety patients, and the answer to “Whether to purchase prepared Chinese drugs” was “No” for prepared prescription (Chinese medicine) and herbal medicines that did not exist in the cost breakdown of the outpatient clinics for anxiety patients. For “Whether to purchase prepared Chinese drugs,” the answer is “NO”; for “Whether to select to have a checkup,” the grouping method is that if there is a checkup fee in the cost breakdown of the outpatient consultation fee for patients with anxiety disorders, the answer is “NO.” For “Whether to select to have a checkup,” the grouping method was that for those who had a checkup fee in the cost breakdown of outpatient visits for patients with anxiety disorders, the answer to “Whether to select to have a checkup” was “Yes”; for those who did not have a checkup fee in the cost breakdown of outpatient visits for patients with anxiety disorders, the answer to “Whether to select to have a checkup” was “NO”; sex is divided into female and male; age is divided into 0–44 years old, 45–59 years old, and 60 years old and above according to the overall trend of rising and then falling in the cost distribution line graph (36); insurance status is divided into urban employees’, urban employees’, urban employees’, and urban employees’ according to the major insurance payment methods in China; insurance status is categorized into urban employees’ basic medical insurance, urban residents’ basic medical insurance, new rural cooperative medical care, self-funded; the type of patient insurance is categorized as medical care, self-funded; hospital level is mainly based on the existing comprehensive rating of the Chinese health care system for the hospital as tertiary hospital, secondary hospital, first-class hospital, unclassified institutions; institution level is mainly based on the existing comprehensive rating of the Chinese health care system for the hospital as tertiary hospital, secondary hospital, first-class hospital, unclassified institutions; and institutions; institution level is mainly based on the geographical location of the current medical and health institutions and the service ability of the strong and weak in China to determine the division into provincial level, municipal level, district level, country level; institution type of medical institutions in accordance with the “Medical Institutions, Health Care, Self-funded”; institution type is divided into general hospital, traditional Chinese medicine hospital, specialized hospital, primary medical institutions according to the Medical Institution Practice License and disease diagnosis and treatment activities.

The most widely used system for accounting for health costs is the System of Health Accounts 2011 (SHA2011), which is the second version of the health cost accounting system jointly revised by several authoritative international organizations in 2011 (37). SHA2011 can be better used to respond to the complex and changing forms and channels of health financing globally, the massive innovation in medical technology and medical knowledge, the increase in data sophistication of health information systems, and policy changes in key populations and diseases. Therefore, this study selects a sample of patients from health care institutions in Liaoning Province from 2015 to 2020, and analyzes different dimensions of costs for outpatients with anxiety disorders from 2015 to 2020 as a way to propose more targeted and informative policy recommendations for anxiety disorders, as well as to promote the progress of research on anxiety disorders in cost analysis.

## Materials and methods

### Data resources

The data and information of this study are obtained from two parts, one part is from the Liaoning Provincial Health and Family Planning Statistical Yearbook, Liaoning Provincial Statistical Yearbook, Liaoning Provincial Financial Annual Report, Liaoning Provincial Government Health Input Monitoring Data, and Liaoning Provincial Social Insurance Fund Accounts from 2015 to 2020, as the total data of Liaoning Province costs, etc.; the other part is from the sample data of the selected medical institutions filled in the consultation information of patients attending the clinic.

### Sampling method

The sampling method adopted in this study was multi-stage stratified random sampling, district sampling based on factors such as the level of economic development and availability of health services in the district. In the first stage, five cities, Dalian, Panjin, Tieling, Fushun and Jinzhou, were selected based on a combination of regional economic development and health resource allocation; in the second stage, one district and two counties were selected in each city, for a total of 15 districts and counties as sample sites; in the third stage, medical institutions were selected in the 15 districts and counties, and the selected medical institutions filled in the consultation information of patients attending the clinic according to the National Center for Health Development Research’s outpatient data template, but a code system was adopted for patients’ personal information, thus discarding obtaining patients’ consent.

### Sample situation

The study discarded the analysis of hospitalization costs due to the small number of hospitalization data entries screened for anxiety disorders, which accounted for less than 1%. From a total of 9,318,513 sample data from 920 healthcare organizations, a total of 109,703 cases of anxiety disorders from 53 sample organizations (5.76%) were screened according to the International Classification of Basic Classification, Tenth Edition (ICD-10) F40-F41 (38), including F40 for phobias (including square phobia: F40.0, social phobia: F40.1, specific phobia: F40.2), F41 for other anxiety disorders (including panic disorder: F41.0, generalized anxiety disorder. F41.1, Mixed anxiety disorder: F41.2-F41.3, Anxiety: F41.8-F41.9) (39). Mental disorders were defined according to a review of the literature according to ICD-10 as F00-F99 (40). The software used for data accounting was STATA 15.0 (Stata Corp, College Station, State of Texas, USA). The cost dates for this study are from January 1, 2015 to December 31, 2020, and since the costs involved in this study are denominated in CNY, there is no unit conversion problem as this study involves costs in Liaoning Province, China. The age range of the study population for anxiety disorders was greater than or equal to 0 years. Gender was categorized as male and female. Information on the characteristics of the study population’s disease included name, ICD-10. And information on costs included total disease outpatient costs, exams, office visits, lab tests, medications, other costs, type of insurance payment, amount paid by insurance, amount paid by the individual in cash. Unclassified institutions for in accordance with the hospital level division standards, is China according to the hospital size, scientific research direction, human resources and technical strength, medical hardware and equipment, etc. on the hospital qualification assessment indicators, in accordance with the “Hospital Classification and Management Standards,” the national unity, regardless of the background of the hospital, all the nature of hospitals, etc., hospitals after the review, determined as unclassified institutions, first-class hospital, secondary hospital, tertiary hospital (41).

### Calculation of CCE

The overall idea of the SHA2011 methodology for accounting for anxiety outpatient costs in Liaoning Province is to use the sample to estimate the whole. This was done by first determining the total health costs in Liaoning Province as the sum of the total costs of public healthcare organizations as determined by the Liaoning Health Financial Yearbook and the total costs of non-public healthcare organizations as determined by the Liaoning Health Statistics Yearbook. Then, based on multi-stage stratified random sampling to obtain the outpatient data of the sample healthcare institutions, and then screening the outpatient data of anxiety disorders of the sample institutions through ICD-10, using the proportion of the outpatient costs of anxiety disorders of the sample institutions to the total outpatient costs of the sample institutions and then multiplying it by the total outpatient costs of Liaoning Province to obtain the total outpatient costs of anxiety disorders in Liaoning Province (42–44). The calculation formula is as follows:


$$S_{\mathrm{OCCE}}=\overset{n}{\underset{i=1}{\sum }}\left(S_{\mathrm{nINC}}+S_{\mathrm{nALL}}\right)$$

S*_OCCE_* is the cost of the anxiety outpatient clinic, S*_nINC_* is the cost of the anxiety outpatient treatment, S*_nALL_* is the anxiety outpatient basic expense benefit, and the anxiety outpatient cost is the sum of the anxiety outpatient treatment cost and the anxiety outpatient basic expense benefit.


$$S_{INC}=S_{\mathrm{TINC}}\times \left(1-\frac{a_{p}}{a}\right)$$

S*_INC_* is the cost of outpatient treatment per person with an anxiety disorder, S*_TINC_* is the total cost of outpatient treatment for anxiety disorders, *ɑ*_p_ is the cost of total preventive services for outpatient treatment for anxiety disorders, and ɑ is the total treatment revenue for anxiety disorders.


$$S_{\mathrm{nINC}}=\overset{n}{\underset{i=1}{\sum }}\left(S_{INC}\times \frac{a_{i}}{a-a_{p}}\right)$$

*ɑ*_i_ is the cost of outpatient treatment services per anxiety disorder patient, and S*_nINC_* is formed by summing the anxiety disorder outpatient treatment costs through each anxiety disorder outpatient.


$$S_{\mathrm{ALL}}=S_{\mathrm{TALL}}-S_{\mathrm{PALL}}$$

S*_TALL_* is the anxiety outpatient total basic expenditure grant, S*_PALL_* is the anxiety outpatient prevention basic expenditure grant, and S*_ALL_* is the anxiety outpatient total treatment basic expenditure grant.


$$S_{\mathrm{nALL}}=\overset{n}{\underset{i=1}{\sum }}\left(S_{\mathrm{ALL}}\times \frac{a_{i}}{a-a_{p}}\right)$$

S*_nALL_* is the sum of the basic expenditure benefit per anxiety outpatient.

### Analysis of factors influencing CCE in outpatient anxiety disorders

Descriptive statistics were analyzed for anxiety disorder outpatient CCE based on different longitudinal groupings (including sex, age, insurance type, etc.), and then one-way analysis was used to determine whether the differences within groups were significant. Since anxiety disorder outpatient costs are skewed data, a logarithmic transformation of outpatient costs to make them normally distributed was performed for multifactor and SEM analysis of factors associated with high anxiety disorder outpatient CCE. Descriptive statistics, univariate analysis, and multifactor analysis were analyzed using IBM SPSS Statistics V.25.0 (IBM Corp), and SEM was constructed and analyzed using AMOS Graphics, V24.0 (SPSS).

## Results

### Summary of anxiety disorder results

More than 90% of anxiety disorder costs occur in outpatient settings, so we only measured CCE for outpatient anxiety disorders. Overall, CCE for anxiety disorders continued to grow from 2015 to 2020 studied, from CNY 99.39 million in 2015 to CNY 233.84 million in 2020. However, CCE for anxiety disorders accounted for more than 15% of CCE for mental disorders of more than 15%, with the lowest percentage of 15.27% in 2015 and the highest percentage in recent years with a value of 18.16% in 2020. The proportion of total health care expenditure (THE) showed a slightly fluctuating trend, leveling off from 2015 to 2017 and gradually increasing from 2018 to 2020. The *per capita* anxiety CCE increases from CNY 2.27 in 2015 to CNY 5.50 in 2020 (Table 1).

**Table 1:** Overall anxiety disorder outpatient CCE, 2015–2020.

Year	Anxiety disorder outpatient CCE (million)	Proportion of mental disorder CCE (%)	Proportion of total health care expenditure (%)	Anxiety CCE *per capita* (CNY)
2015	99.39	16.83	0.09	2.27
2016	102.34	15.27	0.08	2.34
2017	124.86	15.91	0.08	2.86
2018	167.24	17.80	0.11	3.84
2019	202.61	18.13	0.13	4.66
2020	233.84	18.16	0.15	5.50

### Different classifications and costs of anxiety disorders

The anxiety disorders in this study were mainly divided into phobias and other anxiety disorders, and since other anxiety disorders accounted for about 99% of the total, the study was launched on the subdivision of other anxiety disorders, which were mainly divided into panic disorder, generalized anxiety, mixed anxiety disorder, and anxiety, of which the highest outpatient CCE combined in 2015–2020 was generalized anxiety. According to the overall results in Table 1, the outpatient CCE was mainly concentrated in choosing to purchase western drugs, choosing to purchase prepared Chinese drugs, choosing to have a checkup, female, 0–44 years old, self-funded, tertiary hospital, provincial level, general hospital, and 2020. The cost of different types of anxiety disorders varied widely under different subgroups, with a higher proportion of mixed anxiety disorders in choosing to purchase western drugs, choosing to purchase prepared Chinese drugs, tertiary hospitals, provincial level, and 2020; a higher proportion of anxiety in choosing to have a checkup, female, and general hospitals; the highest proportion of phobias in 0–44 years old; and the highest proportion of generalized anxiety disorders in self-funded (Table 2).

**Table 2:** Composition of outpatient CCE for different types of anxiety disorders and different subgroups under them [CNY million (%)].

Variables	Total	Phobias	Other anxiety disorders			
			Panic disorder	Generalized anxiety disorder	Mixed anxiety disorder	Anxiety
Whether to purchase western drugs	930.28 (100.00)	2.44 (100.00)	16.74 (100.00)	709.99 (100.00)	193.02 (100.00)	8.10 (100.00)
Yes	834.69 (89.72)	2.09 (85.76)	16.01 (95.62)	621.58 (87.55)	187.41 (97.09)	7.62 (94.02)
No	95.59 (10.28)	0.35 (14.24)	0.73 (4.38)	88.41 (12.45)	5.62 (2.91)	0.48 (5.98)
Whether to purchase prepared Chinese drugs	930.28 (100.00)	2.44 (100.00)	16.74 (100.00)	709.99 (100.00)	193.02 (100.00)	8.10 (100.00)
Yes	619.92 (66.64)	1.59 (65.15)	8.60 (51.39)	443.61 (62.48)	160.36 (83.08)	5.76 (71.13)
No	310.36 (33.36)	0.85 (34.85)	8.14 (48.61)	266.38 (37.52)	32.66 (16.92)	2.34 (28.87)
Whether to select to have a checkup	930.28 (100.00)	2.44 (100.00)	16.74 (100.00)	709.99 (100.00)	193.02 (100.00)	8.10 (100.00)
Yes	743.92 (79.97)	1.48 (60.88)	12.26 (73.24)	576.85 (81.25)	146.36 (75.82)	6.97 (86.10)
No	186.36 (20.03)	0.95 (39.12)	4.48 (26.76)	133.14 (18.75)	46.67 (24.18)	1.13 (13.90)
Sex	930.28 (100.00)	2.44 (100.00)	16.74 (100.00)	709.99 (100.00)	193.02 (100.00)	8.10 (100.00)
Female	496.06 (53.32)	1.27 (51.58)	8.72 (52.07)	368.78 (51.94)	112.20 (58.13)	5.11 (63.04)
Male	434.23 (46.68)	1.18 (48.42)	8.02 (47.93)	341.20 (48.06)	80.82 (41.87)	2.99 (36.96)
Age	930.28 (100.00)	2.44 (100.00)	16.74 (100.00)	709.99 (100.00)	193.02 (100.00)	8.10 (100.00)
0–44	408.32 (43.89)	1.94 (79.43)	9.78 (58.42)	314.19 (44.25)	79.02 (40.94)	3.40 (41.99)
45–59	273.23 (29.37)	0.46 (18.72)	5.15 (30.76)	211.18 (29.74)	54.02 (27.98)	2.43 (29.98)
≥60	248.73 (26.74)	0.04 (1.84)	1.81 (10.82)	184.61 (26.00)	59.99 (31.08)	2.27 (28.03)
Insurance status	930.28 (100.00)	2.44 (100.00)	16.74 (100.00)	162.30 (100.00)	193.02 (100.00)	8.10 (100.00)
Urban employees’ basic medical insurance	244.88 (26.32)	0.52 (21.25)	6.73 (40.22)	132.31 (18.64)	101.20 (52.43)	4.12 (50.86)
Urban residents’ basic medical insurance	19.73 (2.12)	0.14 (5.60)	0.09 (0.53)	15.64 (2.20)	3.47 (1.80)	0.40 (4.92)
New rural cooperative medical care	2.59 (0.28)	0.00 (0.16)	0.02 (0.09)	2.27 (0.32)	0.28 (0.15)	0.02 (0.22)
Self-funded	663.08 (71.28)	1.78 (72.99)	9.90 (59.15)	559.77 (78.84)	88.07 (45.63)	3.56 (44.00)
Hospital level	930.28 (100.00)	2.44 (100.00)	16.74 (100.00)	709.99 (100.00)	193.02 (100.00)	8.10 (100.00)
Tertiary hospital	924.49 (99.38)	2.40 (98.63)	16.61 (99.25)	704.66 (99.25)	192.79 (99.88)	8.02 (99.00)
Secondary hospital	1.26 (0.14)	0.01 (0.46)	0.04 (0.25)	1.09 (0.15)	0.06 (0.03)	0.06 (0.69)
First-class hospital	0.04 (0.00)	0.00 (0.05)	0.01 (0.07)	0.02 (0.00)	0.01 (0.00)	0.00 (0.00)
Unclassified institutions (41)	4.50 (0.48)	0.02 (0.86)	0.07 (0.43)	4.22 (0.59)	0.16 (0.08)	0.03 (0.31)
Institution level	930.28 (100.00)	2.44 (100.00)	16.74 (100.00)	709.99 (100.00)	193.02 (100.00)	8.10 (100.00)
Provincial level	888.92 (95.55)	2.42 (99.42)	16.71 (99.85)	671.00 (94.51)	192.79 (99.88)	6.00 (74.12)
Municipal level	35.90 (3.86)	0.00 (0.08)	0.01 (0.05)	33.66 (4.74)	0.18 (0.09)	2.04 (25.23)
District level	0.19 (0.02)	0.01 (0.46)	0.01 (0.05)	0.17 (0.02)	0.00 (0.00)	0.00 (0.00)
Country level	5.27 (0.48)	0.00 (0.04)	0.01 (0.04)	5.15 (0.73)	0.06 (0.03)	0.05 (0.65)
Institution type	930.28 (100.00)	2.44 (100.00)	16.74 (100.00)	709.99 (100.00)	193.02 (100.00)	8.10 (100.00)
General hospital	891.54 (95.55)	2.17 (89.03)	15.25 (91.10)	675.23 (95.11)	190.85 (98.88)	8.03 (99.11)
Traditional Chinese medicine hospital	32.77 (3.86)	0.24 (9.94)	1.39 (8.32)	29.70 (4.18)	1.39 (0.72)	0.04 (0.53)
Specialized hospital	1.84 (0.02)	0.01 (0.57)	0.04 (0.21)	1.10 (0.15)	0.67 (0.34)	0.029 (0.35)
Primary medical institutions	4.14 (0.57)	0.01 (0.46)	0.06 (0.36)	3.95 (0.56)	0.11 (0.06)	0.00 (0.00)
Year	930.28 (100.00)	2.44 (100.00)	16.74 (100.00)	709.99 (100.00)	193.02 (100.00)	8.10 (100.00)
2015	99.39 (10.68)	0.17 (7.07)	0.36 (2.17)	87.40 (12.31)	11.45 (5.93)	0.00 (0.00)
2016	102.34 (11.00)	0.05 (1.86)	0.00 (0.01)	89.92 (12.67)	12.13 (6.29)	0.24 (2.96)
2017	124.86 (13.42)	0.33 (13.61)	0.05 (0.32)	100.67 (14.18)	23.11 (11.97)	0.70 (8.60)
2018	167.24 (17.98)	0.70 (28.72)	5.19 (31.02)	126.35 (17.80)	33.76 (17.49)	1.24 (15.26)
2019	202.62 (21.78)	0.53 (21.91)	5.48 (32.71)	145.43 (20.48)	48.93 (25.35)	2.26 (27.86)
2020	233.84 (25.14)	0.65 (26.82)	5.65 (33.77)	160.22 (22.57)	63.64 (32.97)	3.67 (45.32)
Total	930.28 (100.00)	2.44 (0.26)	16.74 (1.80)	709.99 (76.32)	193.02 (20.75)	8.10 (0.87)

### Health financing schemes

**Figure 1:**
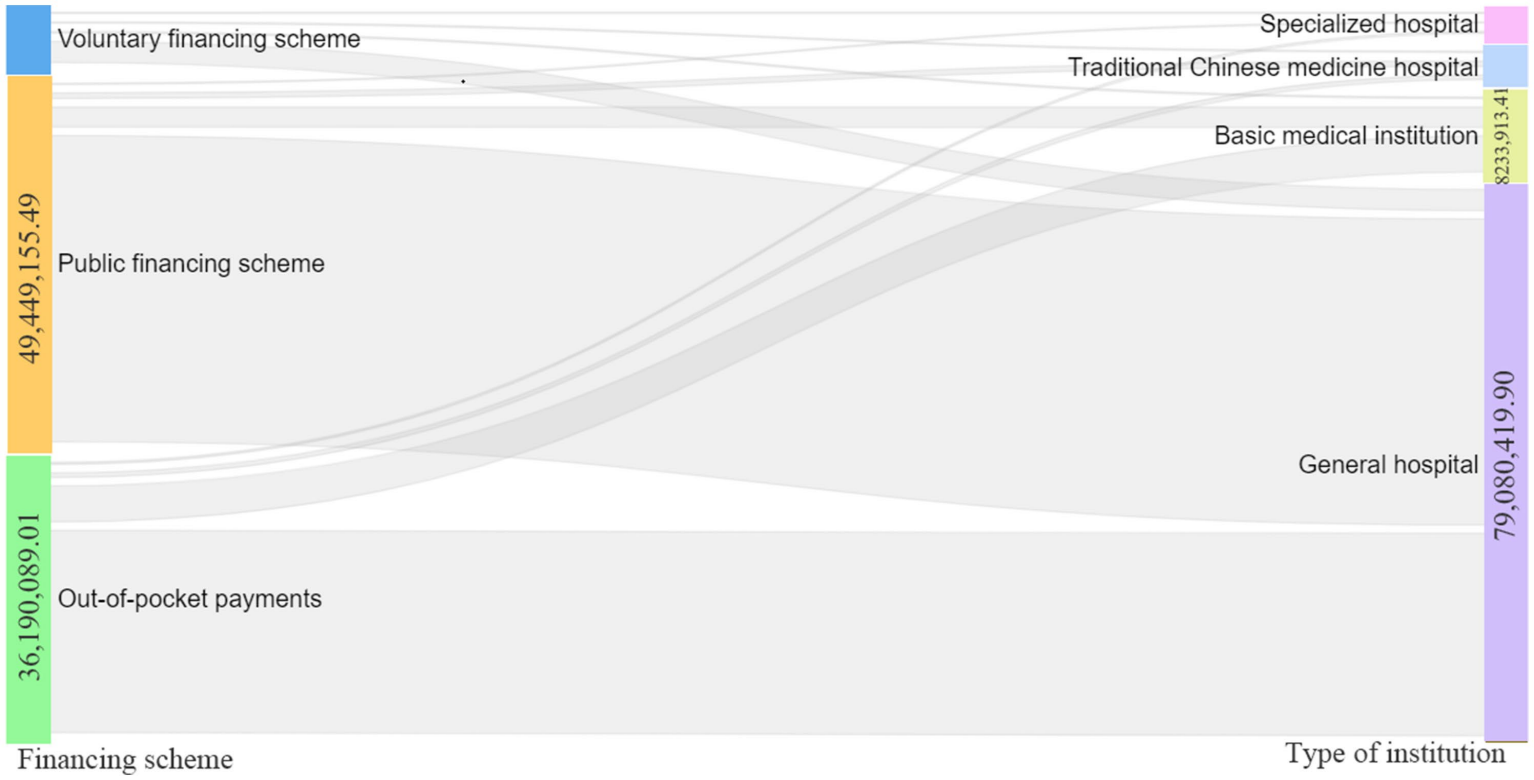
Sankey diagram CCE with 2015–2020 anxiety disorders according to funding structure flow to different institutions average. CCE, current care expenditures.

The main source of financing for anxiety disorders among the three financing methods is public financing (50–60%). In this study, the percentage of public financing shows a trend of increasing and then decreasing, increasing to 62.47% in 2015–2019 and decreasing to 60.97% in 2020, with a continuous increase in costs to CNY 142.57 million in 2015-2020. Public Funding is mainly composed of social health insurance, in line with the overall trend of public funding, with the highest percentage of 58.64% in 2019 and the highest funding value of CNY 131.51 million in 2020. Funding sources are followed by out-of-pocket payments (30–40%). The overall funding value continues to grow to CNY 78.53 million in 2020, in contrast to the trend of public funding share, which gradually decreases to a minimum value of 30.36% in 2015–2019 and rises by 3 percentage points in 2020 (Table 3). From the Sankey diagram (Figure 1), the three types of financing flows to different institutions, the main source of financing for general hospitals comes from public financing (57.96%), followed by out-of-pocket payments (38.23%), and the main source of financing for primary care institutions (including community health service centers, community health service stations, township health centers, outpatient clinics, clinics, and village health offices) Voluntary financing scheme mainly goes to general hospitals (96.08%), but it is also the least source of financing for general hospitals (3.81%).

**Table 3:** Distribution of anxiety outpatient financing costs in Liaoning Province, 2015–2020 [million (%)].

Year	Public financing scheme			Voluntary financing scheme			Out-of-pocket payments	Total
	Total	Social health insurance	Government financing scheme	Total	Social donation	Enterprise financing plan		
2015	51.72 (52.04)	48.67 (48.97)	3.03 (3.07)	8.86 (8.91)	0.00 (0.00)	8.99 (9.05)	38.81 (39.05)	99.39 (100.00)
2016	57.18 (55.87)	50.88 (49.71)	6.45 (6.16)	8.40 (8.21)	0.00 (0.00)	11.47 (11.21)	36.76 (35.92)	102.34 (100.00)
2017	73.53 (58.89)	64.15 (51.38)	11.71 (7.51)	7.78 (6.23)	0.02 (0.01)	7.76 (6.22)	43.55 (34.88)	124.86 (100.00)
2018	100.88 (60.32)	91.39 (54.65)	15.88 (5.68)	11.59 (6.93)	0.15 (0.09)	3.08 (1.84)	54.76 (32.74)	167.24 (100.00)
2019	126.58 (62.47)	118.81 (58.64)	15.74 (3.83)	14.52 (7.17)	0.21 (0.11)	4.18 (2.06)	61.52 (30.36)	202.61 (100.00)
2020	142.57 (60.97)	131.51 (56.24)	25.87 (4.73)	12.74 (5.45)	0.48 (0.20)	2.91 (1.24)	78.53 (33.58)	233.82 (100.00)

### Factors affecting the cost of anxiety disorders

According to the results of the one-way analysis of factors (including Mann–Whitney U test and Kruskal-Wallis H test) in Table 4, the *p*-values are all less than or equal to 0.05, so there is a significant difference in the results of the different subgroups; further two-by-two comparisons of the results and the median comparison show that purchase western drugs, purchase prepared Chinese drugs, have a checkup, male, 45–59 years old, tertiary hospital, provincial level, general hospital, the year of 2017 with two-by-two comparisons within the group p-value are less than or equal to 0.05, and outpatient costs for anxiety disorders had the largest median value, indicating that purchase western drugs, purchase prepared Chinese drugs, have a checkup, male, 45–59 years old, tertiary hospital, provincial level, general hospital, the year of 2017 was associated with high outpatient costs for anxiety disorders.

**Table 4:** Composition of different subgroups and differences in outpatient costs for anxiety disorders in the sample.

Variables	Outpatient expenditure Median (IQR)	Z/H	*p* value	Verify by comparing two-to-two	*p* value
Whether to purchase western drugs		−112.837^a^	<0.001	None	None
Yes	370.35 (121.48–762.27)				
No	76.40 (15.40–278.00)				
Whether to purchase prepared Chinese drugs		−136.02^a^	<0.001		
Yes	732.49 (420.15–1214.53)				
No	207.94 (39.73–488.75)				
Whether to select to have a checkup		−57.266^a^	<0.001		
Yes	411.20 (231.00–782.36)				
No	257.10 (41.10–609.72)				
Sex		−10.277^a^	<0.001		
Female	275.80 (65.40–615.38)				
Male	291.68 (75.40–674.25)				
Age		123.722^b^	<0.001		
0–44	281.35 (69.10–647.43)			(0–44)–(45–59)	<0.001
45–59	286.60 (68.60–641.38)			(0–44)–(≥60)	<0.001
≥60	281.60 (71.60–639.97)			(45–59)–(≥60)	<0.001
Insurance status		1366.34^b^	<0.001	New rural-self	<0.001
Urban employees’ basic medical insurance	367.80 (124.98–757.21)			Employees-residents	<0.001
Urban residents’ basic medical insurance	216.44 (88.39–416.84)			Employees-new rural	<0.001
New rural cooperative medical care	132.40 (72.09–298.75)			Employees-self	<0.001
Self-funded	263.78 (54.40–614.00)			Residents-new rural; residents-self	<0.001
Hospital level		575.414^b^	<0.001	First-secondary	0.221
Tertiary hospital	285.42 (70.60–651.22)			First-unclassified	0.008
Secondary hospital	35.65 (9.00–294.91)			First-tertiary	<0.001
First-class hospital	22.40 (12.40–36.38)			Secondary-unclassified	<0.001
Unclassified institutions	113.26 (87.20–224.33)			Secondary-tertiary; unclassified-tertiary	<0.001
Institution level		4614.347^b^	<0.001	Provincial-municipal	<0.001
Provincial level	308.99 (76.90–692.05)			Provincial-district	<0.001
Municipal level	105.68 (16.50–296.76)			Provincial-country	<0.001
District level	27.20 (12.40–113.20)			Municipal-district; district-country	0.007; 0.003
Country level	110.89 (70.96–239.56)			Municipal-country	1.000
Institution type		1281.681^b^	<0.001	General-traditional	<0.001
General hospital	293.60 (71.10–670.98)			General-specialized	<0.001
Traditional Chinese medicine hospital	183.01 (31.30–362.91)			General-primary	<0.001
Specialized hospital	197.80 (14.16–541.00)			Traditional-specialized	1.000
Primary medical institutions	113.60 (87.20–228.23)			Traditional-primary; specialized-primary	<0.001
Year		1264.284^b^	<0.001	2016–2017	1.000
2015	294.68 (97.10–645.54)			2016–2020	0.050
2016	335.00 (115.00–746.00)			2017–2020	0.721
2017	360.00 (71.00–808.00)			2015–2016; 2015–2017; 2015–2018; 2015–2019	<0.001
2018	258.96 (47.17–565.92)			2015–2020; 2016–2018; 2016–2019; 2017–2018	<0.001
2019	218.31 (38.60–534.84)			2017–2019; 2018–2019	<0.001
2020	315.74 (85.32–728.08)			2018–2019; 2019–2020	<0.001
Total cost	282.03 (70.52–643.54)				

^a^Stands for applying the Mann–Whitney U test (for two independent samples).

^b^Stands for non-parametric Kruskal-Wallis H test (for k independent samples).

IQR means percentile level.

This study had a high linear correlation between the independent variables, the independent variables were not covariate with the dependent variable, and the multivariate analysis model explained 34.8% of the variation in outpatient costs for anxiety disorders in Liaoning Province. According to the standardized coefficient results of the multiple linear regression analysis in Table 5, the high positive effects on high outpatient costs for anxiety disorders were, in order, provincial level, purchase of western drugs, purchase of prepared Chinese drugs, choice of having a checkup, urban employees’ basic medical insurance, 0–44 years old, and the negative effects were, in order, tertiary hospitals, and time of visit for 2018, Traditional Chinese medicine hospital, female, and district level.

**Table 5:** Results of multiple linear regression analysis of outpatient costs for anxiety disorders by different groups.

	Unstandardisation coefficient		Standardization coefficient	*T*	Sig
	B (95%CI)	SE	Beta		
Whether to purchase western drugs					
Yes	0.571	0.004	0.347	136.858	<0.001
No	Reference				
Whether to purchase prepared Chinese drugs					
Yes	0.570	0.004	0.323	129.882	<0.001
No	Reference				
Whether to select to have a checkup					
Yes	0.369	0.006	0.196	76.941	<0.001
No	Reference				
Sex					
Female	−0.013	0.004	−0.009	−3.619	<0.001
Male	Reference				
Age					
0–44	0.106	0.004	0.076	24.789	<0.001
45–59	0.049	0.005	0.032	10.806	<0.001
≥60	Reference				
Insurance status					
Urban employees’ basic medical insurance	0.181	0.004	0.114	43.756	<0.001
Urban residents’ basic medical insurance	0.204	0.011	0.050	18.324	<0.001
New rural cooperative medical care	−0.116	0.026	0.012	4.454	<0.001
Self-funded	Reference				
Hospital level					
Tertiary hospital	−0.892	0.180	−0.157	−4.952	<0.001
Secondary hospital	−0.078	0.056	−0.006	−1.394	0.163
First-class hospital	−0.028	0.143	−0.001	−0.194	0.846
Unclassified institutions	Reference				
Institution level					
Provincial level	1.310	0.174	0.585	7.526	<0.001
Municipal level	0.811	0.174	0.339	4.662	<0.001
District level	−0.067	0.076	−0.003	−0.883	0.377
Country level	Reference				
Institution type					
General hospital	−0.117	0.051	−0.043	−2.300	0.021
Traditional Chinese medicine hospital	−0.196	0.051	−0.065	−3.822	<0.001
Specialized hospital	−0.389	0.059	−0.032	−6.588	<0.001
Primary medical institutions	Reference				
Year					
2015	−0.174	0.007	−0.068	−25.537	<0.001
2016	−0.052	0.007	−0.020	−7.539	<0.001
2017	−0.036	0.008	−0.011	−4.292	<0.001
2018	−0.129	0.005	−0.080	−26.401	<0.001
2019	−0.114	0.005	−0.067	−22.714	<0.001
2020	Reference				

*R*^2^ = 0.348, *p* < 0.001.

B, non-standardized regression coefficient. SE, Standard Error. Beta, standardization regression coefficient. t, *t*-test value (t-statistic). Sig, the meaning of coefficient (*P*).

95%CI, 95% Confidence Intervals.

Taking logarithms of outpatient expenditure in multiple regression analysis.

### Model construction to explore the influencing factors

**Figure 2:**
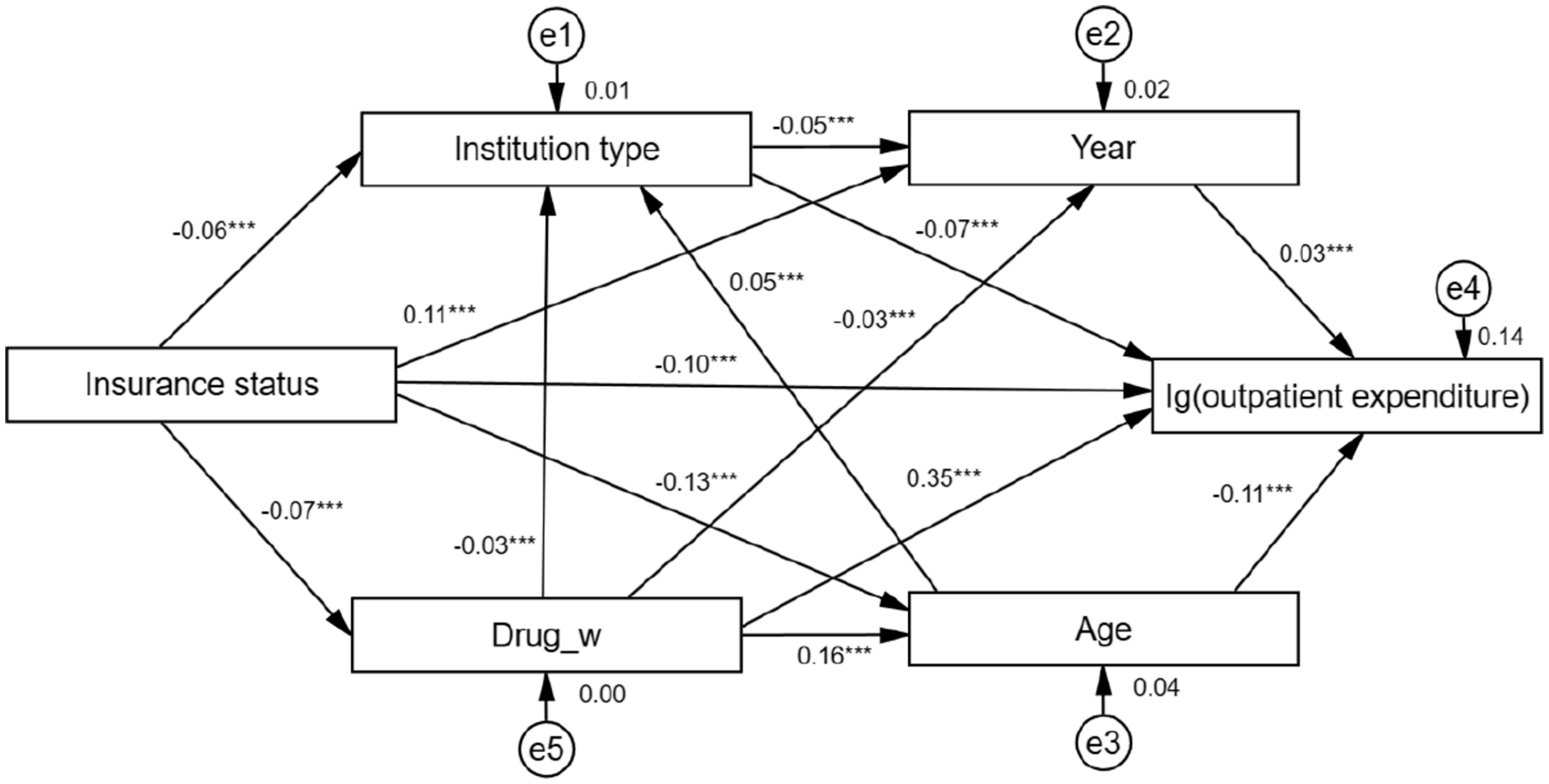
Structural equation modeling of factors influencing anxiety disorder outpatient expenditure. ***Coefficients of the structural equation modeling are of significant meaning.

After constant adjustment and correction the SEM shown in Figure 2 was constructed to explore the factors influencing the outpatient expenditure for anxiety disorders. According to the constant fitting to exclude some variables, the variables included in the model are insurance status, institution type, year, age, and lg (outpatient expenditure), and combined with the reference range of the goodness-of-fit index, it was judged that this model fits well, that is, *χ*^2^ = 0.980, df = 1, *χ*^2^/df = 0.980, CFI = 1.000, NFI = 1.000, RFI = 0.999, IFI = 1.000, TLI = 1.000, RMSEA = 0.000. Insurance status can be determined by institution type (*β* = −0.06, *p* < 0.001), age (*β* = −0.13, *p* < 0.001) and purchase western drugs (drug_w) (*β* = −0.07, *p* < 0.001) negatively and indirectly affect anxiety disorder outpatient expenditure, can positively and indirectly affect anxiety disorder outpatient expenditure through year (*β* = −0.11, *p* < 0.001). Institution type can negatively and indirectly affect anxiety disorder outpatient expenditure through year (*β* = −0.05, *p* < 0.001), and drug_w can indirectly and positively affect anxiety disorder outpatient expenditure through age (*β* = 0.16, *p* < 0.001). Insurance status (*β* = −0.10, *p* < 0.001), and institution type (*β* = −0.07, *p* < 0.001), and age (*β* = −0.11, *p* < 0.001) negatively, year (*β* = 0.03, *p* < 0.001) and drug_w (*β* = 0.35, *p* < 0.001) positively, all directly influenced anxiety disorder outpatient expenditure. This model explained 32.20% of the variation in anxiety disorder outpatient costs.

Insurance status (in order of actual reimbursement ratio): Basic medical insurance for urban workers [actual reimbursement ratio = 75.60% (45)] = 1, urban residents’ basic medical insurance [59.70% (45)] = 2, new rural cooperative medical care [35.00% (46)] = 3, self-funded (0.00%) = 4. Institution type include general hospitals = 1, Chinese hospitals = 2, specialty hospitals = 3, primary health care institutions = 4. Drug_w includes if no western drugs are purchased = 0, purchase of western drugs = 1. Year contain 2015, 2016, 2017, 2018, 2019, 2020. Age: 0–44 = 1.45–59 = 2≥60 = 3.

Taking logarithms of outpatient expenditure in the structural equation model.

## Discussion

This is the first study to measure and analyze the outpatient costs of anxiety disorders in Liaoning Province, China based on SHA2011. Our study provides a certain methodological and theoretical basis for domestic and foreign scholars to assess the economic burden of anxiety disorders, and fills the gap of studying the costs of anxiety disorders based on SHA2011, an international accounting system.

Our study found that funding for the treatment of anxiety disorders comes primarily from public funding programs and out-of-pocket payments, with higher levels in general hospitals, followed by primary care. Out-of-pocket costs are an important correlate of catastrophic healthcare expenditures (47), and if they could be reduced to less than 15%, the likelihood of catastrophic healthcare expenditures for families could be greatly reduced (48). With an out-of-pocket percentage of 35% in this study, families are likely to incur catastrophic medical expenditures and fall into poverty as a result of treating anxiety disorders. And when patients are unable to afford treatment, they are less likely to seek treatment and take medication as prescribed (49), leading to disability and loss of life (50). Therefore, efforts and policies should be focused on reducing OOP costs and their rates (51).

From the point of view of the flow of funds, general hospitals accounted for a large proportion, and primary care organizations accounted for a small proportion. The reasons for this are, firstly, the lack of institutional constraints on patients’ first-visit institutions leads to overuse of excellent health resources and increases the burden of disease (52); and secondly, as patients with anxiety disorders are often accompanied by a variety of physical illnesses, patients are more willing to choose large hospitals with complex laboratory and examination equipment (53). However, anxiety disorders are common in primary care (54–56), only 13% of patients were treated in primary care (57) and 31–41% receive appropriate care for anxiety disorders (58, 59). The main reasons for this are that 84.0 and 86.1% of primary healthcare workers generally have low educational qualifications, resulting in relatively poor service capacity and quality; poor salary and benefits, and more serious brain drain; only basic medicines are allowed to be equipped, with fewer types of medicines, and distribution distances are farther away and inconvenient for transportation, resulting in patients’ medication needs not being met (53); primary care general physicians have limited training in anxiety assessment, evidence-based behavioral and cognitive interventions (58, 60–62).

This study found that among anxiety disorders, generalized anxiety disorder had the highest cost burden, which is consistent with Marciniak’s findings (63). The cost per patient for generalized anxiety disorder was $2,165–3,607 in Europe (64, 65), $6,472 in the United States (66), £1,313 in the United Kingdom (67) and €6,152 in Spain (68). The next most common disorder is mixed anxiety disorder, with 58–59% of patients suffering from both anxiety and depression (69–71), which is the most disabling disorder in the world (72). With an investment of $147 billion in 36 countries to expand coverage of this disorder in 2016–2030 (73). The total cost of mixed anxiety disorders in Spain is about €60 million and the indirect cost is about 40.2 million euros (72); the annual direct medical cost per patient in Singapore is S$1050 (2); the *per capita* social cost of care in the Netherlands is €1,035 (74); the health system, patient, and social costs for Canadian patients to receive minimal appropriate treatment are $5,752, $536, and $6,266, respectively (35); the average annual medical cost of this disorder in the United States cost is $20,963, and the health insurance system pays $9,132 more than without any illness (75). In summary, the burden of treating anxiety disorders is very large.

We explored the factors influencing the cost of outpatient anxiety disorders by univariate analysis, multifactor analysis, and constructing structural equation models. The results of the univariate analysis showed significant differences within each subgroup (*p* < 0.001). Multiple linear regression analysis was used for the multifactor analysis, and the standardized coefficient B for the purchase of western and prepared Chinese drugs was 0.347 and 0.323 higher than that for the absence of western and prepared Chinese drugs, and the SEM results showed the highest path coefficient of 0.35 for the cost of western drugs and log anxiety disorder outpatient costs, indicating that the purchase of drugs was the factor most associated with high anxiety disorder outpatient costs. Some studies have shown that drugs are an effective and more cost-effective first-line treatment option (6) because of its higher dispersion of resources and better patient accessibility (76). Drugs are fundamental to ensuring healing from anxiety disorders (77, 78),with about half of patients with anxiety disorders choosing drugs (approximately $16,855 million) (10, 13) and drugs using increased over time to 63.8% (79), the criterion for adequacy of anxiety disorder treatment is at least 2 months of drugs (80), thus providing evidence that drugs affect a significant factor in high costs. The study found a higher standardized coefficient of 0.196 for selecting to have a checkup than not selecting to have a checkup, suggesting that the choice to have a checkup is associated with high anxiety disorder outpatient costs, which is consistent with the findings of a study in Turkey, which noted that the highest expenditures were for having a checkup (81). A similar finding was found in a study in Sri Lanka, where 14% of the costs in the study were spent on having a checkup (82). The study also found that the standardized coefficient B results from multiple regression analysis showed that urban employees’ basic medical insurance were 0.114 higher than self-funded and the path coefficient between SEM insurance type and log outpatient costs was −0.10, both indicating that urban employees’ basic medical insurance were associated with higher outpatient costs for anxiety disorders, which may be due to the fact that the uninsured consume fewer health care resources and make less use of health care resources, while those with insurance utilize more health resources and therefore have higher health care costs, which is consistent with existing research (83). The standardized coefficient B for age 0–44 years is 0.076 higher than age 60 years, indicating that the young are associated with high anxiety disorder outpatient costs, adolescents are an important part of youth, and some studies have shown that anxiety disorders are the most common psychiatric disorders in adolescence (84), with initial symptoms appearing before school age, with typical anxiety symptoms appearing around age 7 (84), and then a gradual increase in prevalence through adolescence (85), with an average onset age at 13 years (86) and affecting 2.9% of children and 4.6% of adolescents (87). Some studies have shown that the annual *per capita* cost of treating anxiety disorders in adolescents is $6,405, the average cost of using mental health services is $8,615, and the average cost of primary health care is $1,591 (88). Thus the individual and family cost burden of anxiety disorders in adolescence is enormous, and screening, early prevention, and early intervention treatment must focus on this age group in adolescents.

In terms of health care financing and insurance, it is recommended to increase financial investment, raise the proportion of anxiety health investment in government health expenditure, and increase the outpatient reimbursement ratio for anxiety disorders, which can reduce the burden of anxiety treatment for insured people and low-income people; it can also learn from Australia, the United Kingdom, and the Philippines to raise the tax on tobacco to be used for health care financing (52); and learn from the French special tax of 1% on pharmaceutical companies and advertising companies for social security fundraising. At present, the coverage rate of the three basic medical care for urban workers, urban residents and new rural cooperative medical care in China has reached 95%, but there are still many phenomena of poverty due to illness and a large burden of medical care due to illness, which prompts us to establish an integrated medical care policy with consistent rights and responsibilities to realize the integration of the three basic medical care (52). In the choice of institutions, the establishment of grass-roots first diagnosis, hierarchical diagnosis and treatment, two-way referral system of medical care, the first treatment at the grass-roots level, patients who meet the referral conditions are referred to higher-level institutions, and the medical insurance of patients who go beyond the level of treatment will not be reimbursed (52). For primary care institutions, the first step is to improve the psychiatric expertise, medication protocols, and basic cognitive-behavioral therapy knowledge of general practitioners; second, to discuss individualized treatment plans with the patient’s family members (89) and to provide routine psychoeducational and cognitive-behavioral interventions (53); furthermore, and then, to actively integrate video consultations with mental health specialists (90) to reduce the cost of patients’ medical care and the costs outside of the health insurance; Finally, increase primary diagnosis and care by primary care general practitioners; due to the strong emotional confusion and somatic symptoms of anxiety disorders, it is recommended to increase the proportion of primary care facilities less than 1 km away from the population, so as to provide patients with better access to general practitioners (52). Improve the basic drug system and increase the reimbursement ratio of anxiety disorder medication and psychological counseling, because anxiety disorder is a chronic disease that lasts for a long time, so that patients have the ability to afford the cost of anxiety disorder treatment on a continuous basis, which can improve the adherence of patients. Strengthening mental health education for anxiety disorders and other mental health disorders; a large percentage of stigmatized mental illnesses exist in China, carrying stigmatized labels for medical care and treatment (91).

For anxiety disorders, it is best to diagnose and treat them early, so that the prognosis will be better. First of all, in order to enable residents to detect the disease early and receive professional treatment in hospitals at the first time, to reduce the public’s sense of shame from all angles, to guide patients to standardize their medical treatment, and to reduce the medical burden brought by deterioration of the disease, community general practitioners should strengthen the training of knowledge and skills of anxiety disorders and improve the attitude toward patients with anxiety disorders, and strengthen the preventive work, and psychiatrists should also give a certain degree of knowledge of anxiety disorders in the consultation, so that patients can rationally view anxiety disorders and seek medical treatment and prevention earlier. Psychiatrists should also provide some knowledge about anxiety disorders during consultation, so that patients can rationally view anxiety disorders and seek medical treatment and prevention at an earlier stage (90).

This study has some limitations. First, this study only examined direct health care costs regarding anxiety disorders and did not account for economic losses involving other aspects such as lost production, lost work, and lost manpower; second, this study only considered the cost of the first anxiety visit and did not consider the cost of other comorbidities (91). Therefore the above limitations may have contributed to the underestimation of the cost of anxiety disorders in this study. Secondly, as anxiety disorders first develop mostly during adolescence and have associated social and economic costs throughout life (92, 93), schools are one of the key places to provide preventive interventions for anxiety disorders, while being less likely to pose a social risk, are more cost-effective, and, if implemented early, have the potential to prevent the emergence of mental health problems by building resilience and coping strategies in young people (94). Furthermore, exercise can help prevent anxiety symptoms and significantly reduce the risk of anxiety. Exercise can help the body to improve stress capacity and relieve anxiety, and aerobic exercise is more effective in combating anxiety. From early intervention to early prevention, early exercise to reduce psychological stress can prevent people from developing depression and anxiety, thus reducing the incidence of depression and anxiety disorders (95). Finally, residents are more exposed to green spaces, forests or the outdoors, or to blue spaces such as rivers, streams, ponds, lakes and the coast, and for those who live in cities, urban parks can offer similar benefits to forests, and spending time in nature can intervene and reduce the symptoms of anxiety as well as emotional recurrence (96–98).

## Conclusion

This study analyzed the total anxiety disorder outpatient CCE, subtype anxiety disorder costs, funding structure, and specific costs for different subgroups in Liaoning Province from 2015 to 2020 based on SHA2011. Univariate analysis, multi-factor analysis, and construction of SEM on anxiety disorder outpatient costs were used to explore the factors influencing high anxiety disorder outpatient CCE. Finally, actionable recommendations were made in order to reduce the burden of anxiety disorder outpatients.

## Data availability statement

The original contributions presented in the study are included in the article/Supplementary material, further inquiries can be directed to the corresponding author.

## Ethics statement

Patients’ personal information is digitally coded, and the numbers inside are desensitized, so there is no personal privacy involved.

## Author contributions

XS: Conceptualization, Data curation, Investigation, Software, Visualization, Writing – original draft. YZ: Data curation, Investigation, Software, Writing – original draft. QW: Investigation, Methodology, Project administration, Writing – review & editing. PC: Conceptualization, Data curation, Investigation, Software, Supervision, Validation, Writing – review & editing. YM: Funding acquisition, Project administration, Resources, Supervision, Validation, Writing – review & editing.
